# Antibiotic resistance and plasmid analysis of Enterobacteriaceae isolated from retail meat in Lagos Nigeria

**DOI:** 10.1186/s42522-021-00042-x

**Published:** 2021-06-07

**Authors:** Roseline Ekiomado Uzeh, Fadekemisola Adewumi, Bamidele Tolulope Odumosu

**Affiliations:** Department of Microbiology, Faculty of Science, University of Lagos, Akoka-Yaba, Lagos, Nigeria

**Keywords:** Enterobacteriaceae, Plasmids, Antibiotic, Resistance

## Abstract

**Background:**

The presence of antibiotic resistant microorganisms in food is of great concern globally. This research was carried out to detect and characterize plasmid carriage and profiles among members of Enterobacteriaceae from different meat types in Nigeria.

**Method:**

From a total of 80 meat samples comprising of mutton,pork, beef and chicken, organisms belonging to the family Enterobacteriaceae wereisolated by standard procedures and identified by API 20E system. Antibiotics susceptibilities testing (AST) againstselected classes of antimicrobial agents and plasmid extraction was carried outby disc diffusion and alkaline lysis methods respectively.

**Results:**

One-hundred and ten Enterobacteriaceae were isolated,species identification revealed isolates belonging to 7 genera comprising *of Escherichia*, *Enterobacter*, *Klebsiella*,*Citrobacter, Proteus*, *Salmonella *and* Serratia.* Overall resistance of theorganisms to amoxycillin/clavulanic acid was 91 (82.7%), streptomycin 85(75.7%) and perfloxacin 74 (67.2%) while ofloxacin had the highestsusceptibility rate (91.8%). Plasmids profiling revealed ranges of plasmids from1 to 3 copies with estimated sizes range of 700bp to 1.1kb among *E. coli*, *K. pneumoniae*, *E. aerogenes*and *Proteus mirabilis*. All theisolates with plasmids were multidrug resistant and were isolated from chicken excepta strain of *E. coli* from pork whichharboured a single plasmid copy suggesting these meat as reservoirs forantibiotic resistant bacteria.

**Conclusion:**

Our findings revealed high level of meat contamination with antibioticresistant Enterobacteriaceae harbouring resistant plasmids. An integratedsurveillance system and safety practice must be ensured among the processorsand retailers.

## Introduction

Foodborne disease associated with contaminated meats and meat products is a major public health issue. Inadequate food handling practices such as poor sanitation exercise, weak or poor safety laws, and regulatory system enforcements, lack of enlightenment, and infection awareness are some of the major factors promoting foodborne diseases in developing countries [1]. The microbiological quality of any meat depends on the health and physical status of the animal at the point of slaughter, handling, environmental hygiene, and storage [2].

The family Enterobacteriaceae consists of a large heterogeneous group of Gram-negative rod-shaped bacteria that are naturally found in the mammalian gut although can also be found in other environments. They are useful indicators of food quality, hygiene, and contamination, examples include *Escherichia coli, Salmonella* spp. and *Shigella* spp. Enterobacteriaceae is responsible for a range of enteric infections such as diarrhea and endocarditis, to infections of the respiratory tract, skin, soft-tissues, urinary tract, joints, bones, eyes and central nervous system [3].

Contamination of meat and meat products by antibiotic-resistant pathogenic members of Enterobacteriaceae is of great concern globally because of the health consequences which in some cases result in high mortality. Reports of elevated antibiotic resistance and dissemination among these foodborne pathogens have also been previously reported [4, 5]. Antibiotic use in livestock production is sometimes unavoidable because of the treatment of infections caused by different microorganisms which may not be readily preventable due to lack of vaccines. It is however true that these antibiotics are sometimes given at a sub-therapeutic dosage and this often leads to selective pressure and proliferation of antimicrobial resistance and spread among the animal intestinal flora. *Escherichia coli* and other members of Enterobacteriaceae have been described with high proficiency for transmission of antibiotic resistance genes via mobile genetic elements such as plasmids and integrons to other intestinal organisms [6].

Meat is a nutrient-rich food with a vital amount of proteins, vitamins and minerals as well as great bioavailability than most other foods [7]. It is highly consumed in Nigeria even though it has been recognized as one of the main vehicles responsible for the transmission of foodborne pathogens to humans [8]. The initial exposure of meat to the gut content of food animals during slaughtering serves as a predisposing factor for meat contamination with members of Enterobacteriaceae and subsequently, during processing and post-process while on retail, from environmental sources and handlers. Considering foodborne outbreaks by pathogenic members of Enterobacteriaceae vis a viz. pathogenic strains of *Escherichia coli*, *Salmonella*, *Shigella, Klebsiella*, etc. it becomes imperative to continuously carry out surveillance of meat contamination by pathogenic organisms. Data obtained from the contamination of meat by Enterobacteriaceae and their antibiotic resistance profile will be valuable for the logical assessment of the safety of meat as to well as elucidate further, the possible transmission of antibiotic resistant foodborne pathogens through contaminated meat to consumers.

The present study aimed to detect members of Enterobacteriaceae in locally processed meat samples and to investigate the presence of plasmids as a mode of antibiotic resistance gene transmission among them.

## Materials and methods

### Sample collection

A cross-sectional study was conducted on processed retail meat to determine the bacteriological quality and antibiotic susceptibility of Enterobacteriaceae in the Lagos metropolis. The sampling criterion applied was the simple random sampling method. A total of 80 samples of retail meat [comprising of mutton (*n* = 20), pork (*n* = 20), beef (*n* = 20), and chicken (*n* = 20)] were randomly purchased from retail points at Agege, Obalende, Mushin, and Bariga (sampling areas) in Lagos Metropolis from March to June 2019. The sampling areas were randomly selected from the two major zones that constitute Lagos State- Lagos Mainland and Lagos Island. The meat samples were collected in Ziploc bags and immediately transported to the laboratory for microbiological analysis.

### Sample analysis

All meat samples were assayed for the presence of any member of Enterobacteriaceae by weighing 25 g of each meat sample aseptically and added to 225 ml of sterile 0.1 % buffered peptone water and blended for 2 min. in sterile stomacher bag [9]. Tenfold serial dilutions of up to 10^6^ were made from the homogenized sample and 1ml from each final dilution was plated on Petri dishes containing different agars of MacConkey, Eosin Methylene Blue, *Salmonella-Shigella* and incubated for a minimum of 24 h until visible growths were observed. Isolates were subculture based on their phenotypic appearances and colonial morphologies, for instance, isolates that appeared on MacConkey agar (MCA) as lactose and non-lactose fermenters, were subculture separately on different MCA and Salmonella-Shigella agar (SSA). Colonies that appeared as dark centered colonies and those with green metallic sheen were picked and subculture on SSA and Eosin methylene blue agar (EMBA) respectively and subsequently screened on sorbitol MacConkey agar (SMAC) as described [9].

### Identification of isolates

All pure cultures of suspected members of Enterobacteriaceae were subjected to a preliminary standard biochemical test for identification. Presumptively identified members of Enterobacteriaceae were further screened by using API 20E system (Bio-Merieux, France) according to manufacturer’s instructions.

### Antimicrobial susceptibility testing

The antibiotics susceptibility of the isolates against commonly prescribed drugs for enteric and foodborne infections was determined and interpreted by standard procedures and guidelines for the disc diffusion method [10]. The following antibiotics were used; trimethoprim/sulfamethoxazole (25 µg), chloramphenicol (30 µg), ciprofloxacin (10 µg), amoxicillin (30 µg), amoxicillin/clavulanic acid (20/10µg), gentamicin (10 µg), pefloxacin (30 µg), ofloxacin (10 µg), streptomycin (30 µg).

### Plasmid DNA extraction

The plasmid extraction was carried out as previously described [11] with little modifications using 100 bp and 1 kb plasmid ladders. Twenty-five (*n* = 25) isolates were picked at random among the antibiotics resistant isolates for plasmid investigation. Briefly, bacterial cultures were grown in nutrient broth with an optimized concentration of antibiotics at 37 °C overnight in a shaker incubator at 150 rpm. After harvesting, the culture was transferred to a 1.5-ml Eppendorf tube containing a lysis buffer, heated for 15 min at 70^o^C, mixed with an equal volume of phenol: chloroform: isopropanol (25:24:1) and afterward centrifuged. The supernatants were collected using a pipette and added to a blue loading dye and run on 1 % agarose gel Tris–acetate–EDTA buffer for an hour at 80 V. plasmid bands were visualized using Gel Documentation system and ultraviolet light transilluminator.

### Statistical analysis

A one-way analysis of variance (ANOVA) test was used to compare the difference in the prevalence of isolates recovered from the various categories of samples with a significant level at *p* < 0.05.

## Results

A total of 110 isolates of members of Enterobacteriaceae were obtained from the 80 meat samples analyzed. The colonial morphology, Gram reaction, and results obtained from the API 20E kits revealed the identity of the isolates as *Enterobacter aerogenes, E. cloacae, Citrobacter freundii, Proteus mirabilis, Klebsiella pneumonia, K. planticola, Salmonella* spp., *E. coli*, and *Serratia odorifera.* The frequency of occurrence of these organisms in the meat samples is as shown in Table 1 with *Enterobacter* spp. having the highest frequency of occurrence of 26 (23.6 %) and *Serratia* spp. having the lowest 3 (2.7 %).

**Table 1:** Frequency of occurrence of members of Enterobacteriaceae in locally processed meat

Enterobacteriaceae	Beef	Pork	Chicken	Mutton	Total Frequency (%)
***Proteus*** **spp.**	5	2	0	4	11 (10)
***Enterobacter*** **spp.**	9	13	2	2	26 (23.6)
***Citrobacter*** **spp**.	2	0	7	5	14 (12.7)
***Escherichia coli***	8	9	4	2	23 (21)
***Serratia*** **spp**.	0	2	1	0	3 (2.7)
***Klebsiella*** **spp**.	5	7	4	2	18 (16.4)
***Salmonella*** **spp**.	0	5	5	5	15 (13.6)
**Grand Total**	29	38	23	20	110 (100)
**Standard Deviations**	3.6253	4.5774	2.43	1.8645	3.6253

### Antibiotics susceptibility test

The antibiotics resistance profile of the organisms is presented in Table 2. Ninety-one (82.7 %) of Enterobacteriaceae isolated were resistant to amoxicillin/clavulanic acid while 85 (75.7 %) were resistant to both amoxicillin and streptomycin, 74 (67.2 %) were resistant to perfloxacin while 65 (59.1 %) showed resistance to both sparfloxacin and ciprofloxacin. A similar resistance level of 45 (40.9 %) was found for both gentamicin and trimethoprim/sulfamethoxazole while 91.8 % of the organisms were susceptible to ofloxacin.

**Table 2:** Resistance of enteric bacteria to antibiotics

Enterobacteriaceae		Number of resistant strains (Percentage Resistance)									
	**Total No.**	AMX	AUG	GEN	CHL	SPX	TIM	CIP	OFX	STR	PEF
*Proteus* spp.	11	11(100)	9(81.8)	10(90.9)	10(90.9)	7(63.6)	9(81.8)	6(54.5)	0(0.0)	11(100)	10(90.9)
*Enterobacter* spp.	26	14(53.8)	26(100)	15(57.7)	0(0.0)	13(50)	0(0.0)	15(58.8)	0(0.0)	7(26.9)	20(76.9)
*Citrobacter* spp.	14	7(50)	13(92.9)	6(42.9)	12(85.7)	8(57.1)	0(0.0)	9(64.3)	7(50)	14(100)	5(35.7)
*Serratia* spp.	3	3(100)	3(100)	1(33.3)	0(0.0)	0(0.0)	2(66.7)	0(0.0)	0(0.0)	0(0.0)	1(33.3)
*Klebsiella* spp.	18	15(83.3)	2(11.1)	3(16.7)	1(5.6)	15(83.3)	11(61.1)	17(94.4)	0(0.0)	16(88.8)	18(100)
*Salmonella* spp.	15	12(80)	15(100)	10(83.3)	12(80)	11(61.1)	0(0.0)	10(55.5)	0(0.0)	14(93.3)	10(55.5)
*E. coli*	23	23 (100)	23 (100)	0 (0.0)	13(37.5)	8(56.5)	23(100)	2 (8.6)	2(8.6)	23(100)	5 (21.7)
Total	110	85(77.2)	91(82.7)	45(40.9)	48(43.6)	65(59.1)	45(40.9)	65(59.1)	9 (8.18)	85(77.3)	74(67.2)

*AM* Amoxicillin (30ug), *AMC* Amoxycilin/clavulanic acid (20/10ug), *GEN* Gentamicin (10ug), *CHL* Chloramphenicol (30ug), *SPX *Sparfloxacin (10ug), *TIM* trimethoprim/sulfamethoxazole (30ug), *CIP* Ciprofloxacin (10ug), *OFX* ofloxacin (10ug), *STR *Streptomycin (10ug), *PEF* Pefloxacin (30ug)

At genera level, complete resistance (100 %) was observed among *Proteus* spp. against amoxicillin and streptomycin, *Enterobacter* spp. showed complete resistance to amoxicillin/clavulanic acid, so were *E. coli*, *Serratia* spp. and *Salmonella* spp. There was 100 % resistance by *E. coli* to trimethoprim/sulfamethoxazole, amoxicillin, and streptomycin. *Klebsiella* spp. and *Citrobacter* spp. showed complete resistance to pefloxacin and streptomycin respectively. In terms of susceptibilities, there was 100 % susceptibility to chloramphenicol, sparfloxacin, ciprofloxacin, ofloxacin, and streptomycin by *Serratia* spp. *Enterobacter* spp. were completely susceptible to chloramphenicol, ofloxacin, and trimethoprim/sulfamethoxazole, while *E. coli* and *Salmonella* spp. were completely susceptible to gentamicin, trimethoprim/sulfamethoxazole, and ofloxacin respectively.

### Plasmid analysis

Nine (36 %) out of 25 isolates harboured plasmids ranging from 1 to 3 copies with an estimated size range of 700 bp to 1.1 kb (data not shown), and the highest number of plasmids 4 (44.44 %) was detected in *E. coli*, followed by *K. pneumonia* and *E. aerogenes* both having 2 (22.22 %) each and *P. mirabilis* 1 (11.11 %). A maximum of 3 copies of plasmids was found in *E. coli* isolated from chicken while the remaining isolates had 1 copy each. All the isolates with plasmids were from chicken except a strain of *E. coli* from pork harboring a single plasmid copy.

### Statistical analysis

According to the statistical analysis, the *f*-ratio value is 0.82804 while the *p*-value is 0.491442 hence there was no significant difference in the incidence of Enterobacteriaceae in pork, beef, mutton, and chicken at *p* < 0.05 significant level.

## Discussion

 In this study, Enterobacteriaceae were isolated from locally processed beef, pork, mutton, and chicken on retail. Overall, there was no significant difference in Enterobacteriaceae contamination levels among the different meat types, however, the highest number of isolates 38 (34.5 %) recovered was from pork and *Enterobacter* spp. dominated the overall population of the isolates recovered as 23 % of its species was isolated from different meat samples investigated followed by *E. coli* 21 % while *Serratia* spp. was the least isolated from all the meat (Table 1).

The source of the Enterobacteriaceae isolated from the meat in this study may have been from contaminated processing water, contact surfaces, and food handlers who may have compromised hygiene practices. Previous studies have documented the presence of one or more members of Enterobacteriaceae from retail meat and carcasses of animals. Uzeh and Agunlanna [12] detected *E. coli* O157:H7 and other *E. coli* strains in 37 and 63 % respectively of meat samples from different parts of cattle carcass. In agreement with the results of this study, although at varying prevalence, Enterobacteriaceae have been previously reported from different retail meat samples. Studies from Egypt documented the presence of *Proteus* spp., *E. coli Citrobacter* spp. and *Klebsiella* spp. in retail meats from sellers [13], *Klebsiella* spp., *Serratia* spp., *E. coli*, *Enterobacter* spp., *Citrobacter* spp., *Proteus mirabilis* and *Serratia* spp., were also reported from retail beef and poultry in the United State [14]. *Salmonella* isolates of 10 (2.3 %) were obtained from retail beef and related meat products in Zaria, Nigeria [15], a value lower than the 15 (13.6 %) obtained in the present study. The variation in result may have been due to the different meat products investigated in both studies. *Salmonella* is one of the most important foodborne pathogens in the world, its isolation from locally processed meats in Nigeria is worrisome and calls for more urgent attention.

Enterobacteriaceae isolated in this study showed high levels and multiple resistance traits to antibiotics investigated, which can be transmitted from food products to humans via consumption. Out of all the antibiotics investigated for activities against these pathogens, ofloxacin remained the most potent with an overall susceptibility rate of 91.8 % by the organisms. When compared to other members of fluoroquinolones investigated, ofloxacin showed remarkably higher efficacy than ciprofloxacin and sparfloxacin both with equal overall 40.9 % susceptibility rate, and pefloxacin with 32.8 % susceptibility rates. Ofloxacin’s higher antimicrobial activities have been attributed to the presence of alkylated piperazine group at one of its structural positions [16].

Diverse rates of resistance to the different classes of antibiotics were observed in this study and it ranged from 5.6 to 100 % with resistance to streptomycin, trimethoprim/sulfamethoxazole, amoxicillin, and amoxicillin/clavulanic acid is most frequently observed. The observed resistance to antibiotics in this study is so disturbing considering the fact that most of the antibiotics investigated are not commonly used in livestock production but are rather prescribed against human infections caused by enteric bacteria. These results are in agreement with previous related studies that reported similar high resistance to the above antimicrobial agents [17]. A high level of resistance or complete resistance (100 %) to and amoxicillin/clavulanic acid was observed by 85 % of the isolates in this study. Both drugs belong to beta-lactam and beta-lactamase inhibitor classes of antibiotics respectively and are commonly prescribed for infections associated with Gram negative bacteria. Resistance to these two classes of antibiotics is usually mediated by beta-lactamase enzymes encoded on genes borne on mobile genetic elements such as plasmids and integrons that amoxicillin enable them to efficiently hydrolyze these drugs [18, 19].

Enterobacteriaceae are well known for harbouring plasmids in multiple copies of varying sizes [20]. The occurrence of plasmids among the resistant Enterobacteriaceae in this study was detected among 9 out of 25 isolates investigated, accounting for 36 % of the total Enterobacteriaceae investigated for plasmids. Isolates positive for the presence of plasmid harboured up to 3 copies of the extra-chromosomal DNA with estimated sizes of up to 1.1 kb. The findings from the present study are in agreement with previous studies on the carriage of plasmids by members of Enterobacteriaceae [20, 21]. There was a correlation between plasmid profiles among the isolated bacteria and their multiple drug resistance in the present study (Table 3), thus suggesting the carriage of plasmids may be associated with the observed resistance profiles in this study. Although one of our study limitation is the inability to provide an evidence of a link between the presence of plasmids and the antimicrobial resistance profiles observed. Antibiotic resistant genes encoding multiple resistance to antibiotic classes such as fluoroquinolones, aminoglycosides and beta-lactams borne on plasmids are capable of bacteria to bacteria transmission via horizontal gene transfer among bacterial communities [22, 23] Interestingly, carriage of plasmids among the selected isolates was more frequent in bacteria isolated from chicken indicating chicken meat may be a reservoir of antimicrobial resistance as previously opined elsewhere [24].

**Table 3:** Plasmid Profiles of 9 Enterobacteriaceae isolated from various meat sources

Source	Isolates	Plasmid sizes (kb)	Plasmid copies	Resistance profile
Chicken	*E.coli* BCI15	0.7, 0.75, 1.1	3	AMX, AUG, CHL, SPX, TIM, CIP, OFX, STR, PEF
Chicken	*K. pneumoniae* BCI28	0.7, 0.9	2	AMX, AUG, CHL, SPX, TIM, CIP, STR, PEF
Chicken	*K. pneumoniae* ACI77	0.75, 0.9	2	AMX, AUG, GEN, SPX, TIM, CIP, STR, PEF
Chicken	*E. aerogenes* OCI46	0.7, 0.8	2	AMX, AUG, GEN, SPX, CIP, STR, PEF
Chicken	*E. aerogenes* ACI12	0.7, 0.75	2	AMX, AUG, GEN, SPX, CIP, STR, PEF
Chicken	*E.coli* ACI22	0.8	1	AMX, AUG, CHL, SPX, TIM, CIP, STR, PEF
Chicken	*E.coli* OCI50	0.8	1	AMX, AUG, CHL, SPX, TIM, STR, PEF
Chicken	*P. mirabilis* BCI08	0.7	1	AMX, AUG, GEN, CHL, SPX,TIM, CIP, STR, PEF
Pork	*E.coli* API33	0.75	1	AMX, AUG, CHL, SPX, TIM, STR, PEF

In conclusion, the data presented in this study shows that locally processed pork, beef, mutton, and chicken on retail may contribute significantly to the spread of antibiotic-resistant Enterobacteriaceae to the community. The occurrence of antibiotic resistant Enterobacteriaceae in these retail meats is a potential risk to public health. It is also clear from this study that certain levels of safety practices that help to minimize food contaminations may have been compromised during meat processing and retail. Measures should therefore be put in place to reduce the proliferation of pathogenic antibiotic resistant bacteria in these meat products.

## Data Availability

The datasets used and analyzed during the current study are available from the corresponding author on reasonable request. All data generated or analyzed during this study are also included in this published article

## Abbreviations

AST: Antibiotics susceptibilities testing
API 2: Analytical Profile Index
MCA: MacConkey agar
SSA: Salmonella-Shigella agar
EMBA: Eosin methylene blue agar
ANOVA: One-way analysis of variance
